# Kidney damage and associated risk factors in rural and urban sub-Saharan Africa (AWI-Gen): a cross-sectional population study

**DOI:** 10.1016/S2214-109X(19)30443-7

**Published:** 2019-12

**Authors:** Jaya A George, Jean-Tristan Brandenburg, June Fabian, Nigel J Crowther, Godfred Agongo, Marianne Alberts, Stuart Ali, Gershim Asiki, Palwende R Boua, F Xavier Gómez-Olivé, Felistas Mashinya, Lisa Micklesfield, Shukri F Mohamed, Freedom Mukomana, Shane A Norris, Abraham R Oduro, Cassandra Soo, Hermann Sorgho, Alisha Wade, Saraladevi Naicker, Michèle Ramsay, AWI-Gen and the H3Africa Consortium

**Affiliations:** Department of Chemical Pathology (J A George PhD, Prof N J Crowther PhD) and Division of Human Genetics (G Agongo MPhil, P R Boua MSc, Prof M Ramsay PhD), National Health Laboratory Service and University of Witwatersrand, Johannesburg, South Africa; Sydney Brenner Institute for Molecular Bioscience (J-T Brandenburg PhD, G Agongo, S Ali PhD, P R Boua, F Mukomana MSc, C Soo MSc, Prof M Ramsay), Wits Donald Gordon Medical Centre (J Fabian MD), School of Clinical Medicine (J Fabian, Prof S Naicker PhD), School of Pathology (G Agongo, P R Boua, Prof M Ramsay), MRC/Wits Rural Public Health and Health Transitions Research Unit (Agincourt), School of Public Health (F X Gómez-Olivé PhD, A Wade DPhil), and MRC/Wits Developmental Pathways for Health Research Unit (L Micklesfield PhD, Prof S A Norris PhD), Faculty of Health Sciences, University of the Witwatersrand, Johannesburg, South Africa; Navrongo Health Research Centre, Navrongo, Ghana (G Agongo, A R Oduro MBChB); Department of Pathology and Medical Science, School of Health Care Sciences, Faculty of Health Sciences, University of Limpopo, Polokwane, South Africa (Prof M Alberts PhD, F Mashinya PhD); African Population and Health Research Center, Nairobi, Kenya (G Asiki PhD, S F Mohamed MPH); and Clinical Research Unit of Nanoro, Institut de Recherche en Sciences de la Sante, Nanoro, Burkina Faso (P R Boua, H Sorgho PhD)

## Abstract

**Background:**

Rapid epidemiological health transitions occurring in vulnerable populations in Africa that have an existing burden of infectious and non-communicable diseases predict an increased risk and consequent prevalence of kidney disease. However, few studies have characterised the true burden of kidney damage and associated risk factors in Africans. We investigated the prevalence of markers for kidney damage and known risk factors in rural and urban settings in sub-Saharan Africa.

**Methods:**

In this cross-sectional population study (Africa Wits-International Network for the Demographic Evaluation of Populations and their Health Partnership for Genomic Studies [AWI-Gen]), we recruited unrelated adult participants aged 40–60 years from four rural community research sites (Nanoro, Burkina Faso; Navrongo, Ghana; Agincourt and Dikgale, South Africa), and two urban community research sites (Nairobi, Kenya; and Soweto, South Africa). Participants were identified and selected using random sampling frames already in use at each site. Participants completed a lifestyle and medical history questionnaire, had anthropometric and blood pressure measurements taken, and blood and urine samples were collected. Markers of kidney damage were defined as low estimated glomerular filtration rate (eGFR; <60 mL/min per 1·73 m^2^), presence of albuminuria (urine albumin creatinine ratio >3 mg/mmol); or chronic kidney disease (low eGFR or albuminuria, or both). We calculated age-adjusted prevalence of chronic kidney disease, low eGFR, and albuminuria by site and sex and used logistic regression models to assess risk factors of kidney damage.

**Findings:**

Between August, 2013, and August, 2016, we recruited 10 702 participants, of whom 8110 were analysable. 4120 (50·8%) of analysable participants were male, with a mean age of 49·9 years (SD 5·8). Age-standardised population prevalence was 2·4% (95% CI 2·1–2·8) for low eGFR, 9·2% (8·4–10·0) for albuminuria, and 10·7% (9·9–11·7) for chronic kidney disease, with higher prevalences in South African sites than in west African sites (14·0% [11·9–16·4] in Agincourt *vs* 6·6% [5·5–7·9] in Nanoro). Women had a higher prevalence of chronic kidney disease (12·0% [10·8–13·2] *vs* 9·5% [8·3–10·8]) and low eGFR (3·0% [2·6–3·6] *vs* 1·7% [1·3–2·3]) than did men, with no sex-specific differences for albuminuria (9·9% [8·8–11·0] *vs* 8·4% [7·3–9·7]). Risk factors for kidney damage were older age (relative risk 1·04, 95% CI 1·03–1·05; p<0·0001), hypertension (1·97, 1·68–2·30; p<0·0001), diabetes (2·22, 1·76–2·78; p<0·0001), and HIV (1·65, 1·36–1·99; p<0·0001); whereas male sex was protective (0·85, 0·73–0·98; p=0·02).

**Interpretation:**

Regional differences in prevalence and risks of chronic kidney disease in sub-Saharan Africa relate in part to varying stages of sociodemographic and epidemiological health transitions across the area. Public health policy should focus on integrated strategies for screening, prevention, and risk factor management in the broader non-communicable disease and infectious diseases framework.

**Funding:**

National Human Genome Research Institute, Office of the Director, Eunice Kennedy Shriver National Institute of Child Health and Human Development, National Institute of Environmental Health Sciences, the Office of AIDS Research, and National Institute of Diabetes and Digestive and Kidney Diseases, all of the National Institutes of Health, and the South African Department of Science and Technology.

## Introduction

In sub-Saharan Africa, infectious diseases are the most common causes of death and years of life lost; however, the relative contribution of non-communicable diseases is increasing.^1^ The emergence of non-communicable diseases in sub-Saharan Africa reflects complex sociodemographic transitions characterised by improved survival into adulthood with relative ageing of populations, rapid urbanisation, and changes in diet, levels of activity, and habits such as increased smoking and alcohol consumption.^2^ Chronic kidney disease is the final common pathway for many infections and non-communicable diseases and is an independent risk factor for death from cardiovascular causes, leading to growing concern regarding increases in the estimated global prevalence, ranging from 8% to 16%.^3^ Two recent systematic reviews of chronic kidney disease in sub-Saharan Africa high lighted the paucity of reliable population-based data on the prevalence of chronic kidney disease, with weaknesses in study design, laboratory methods for creatinine measurement, and the absence of standardised criteria for the definition of chronic kidney disease being cited as reasons.^4,5^

In this study, we determined population-based prevalence estimates of kidney disease and investigated risk factors for kidney disease in Africans from rural and urban areas in South Africa and countries in west and east Africa. This is a substudy of the Africa Wits-International Network for the Demographic Evaluation of Populations and their Health (INDEPTH) Partnership for Genomic Studies (AWI-Gen), which includes five participating health and demographic surveillance sites that are part of the INDEPTH network. The aim of the broader AWI-Gen study is to determine population prevalences of cardiometabolic diseases, understand associated risk factors and differences in regional burden, and explore gene–gene and gene–environment interactions that contribute to disease risk in sub-Saharan Africa.

## Methods

### Study design and participants

Details of the data collection methods of AWI-Gen have been published.^6^ In summary, this is a population-based cross-sectional study that recruited unrelated participants aged 40–60 years from six study sites in four participating countries in sub-Saharan Africa. The sites were community research centres based in Nanoro, a rural community in Burkina Faso; Navrongo, a rural district in Ghana; Agincourt and Dikgale, both semi-rural areas with clustered villages in northern South Africa; two urban slums in Nairobi, Kenya; and Soweto, an urban region in South Africa. Participants were identified and recruited by use of random sampling using existing sampling frames in use at each study site, with slight deviations at Agincourt and Soweto. In Agincourt, individuals were recruited who had previously consented to participate in earlier studies for which they had been randomly selected, and random sampling from the rest of the community was done to increase the sample size. In Soweto, 700 women had participated in a previous study (SWEET)^7^ for which they were randomly selected from an existing cohort (Birth to 20+).^8^ The remaining study population from Soweto was identified and recruited from the men and women in the community. The site in Dikgale used convenience sampling based on eligible participant availability.^6^ At all sites participants were excluded if they were pregnant women, closely related to another participant (ie, first degree relatives), or a recent immigrant (<10 years) to the community.

This study was approved by the Human Research Ethics Committee (Medical) of the University of the Witwatersrand (protocol numbers M121029 and M170880), and each contributing study site obtained additional local ethics approval, as required. Additionally, each research site sought approval from their local ethics review board before commencing any participant-related activities.^6^ Participants were required to give written or verbal informed consent before study activities.

### Data collection and definitions

Trained field workers, nurses, or physicians took measurements of participants. Weight and height of participants while wearing light clothes and barefoot were measured using a calibrated electronic scale (to the nearest 0·1 kg; Physician Large Dial 200 kg capacity scales, Kendon Medical, South Africa) and stadiometer (Harpenden digital stadiometer, Holtain, Wales, UK). Body-mass index (BMI) was calculated (kg/m^2^). Waist circumference was measured using a stretch-resistant tape (SECA, Hamburg, Germany) placed around the narrowest part of the torso, halfway between the iliac crest and the lowest rib, and hip circumference around the widest part of the buttocks.

Venous blood was drawn after an overnight fast, processed for serum, plasma, and buffy coats and stored at −80°C before analysis at a central laboratory in Johannesburg, South Africa. A spot sample of urine was collected and stored at −80°C and sent to the central laboratory. Glucose concentration was measured using a glucose oxidase method; triglycerides, total cholesterol, and HDL cholesterol were measured enzymatically; and LDL cholesterol was calculated using the Friedewald formula. Urinary albumin concentration was measured with immunoturbidimetry and creatinine concentration was measured with Jaffe’s kinetic method, both using a urine sample that had been stored at −80°C. The urinary albumin to creatinine ratio (ACR; mg/mmol of creatinine) was calculated from these measurements. Serum creatinine assays were done with an isotope dilution mass spectrometry (IDMS) traceable Jaffe method.

Estimated glomerular filtration rate (eGFR) was calculated using four different estimates: the 4-variable Modification of Diet in Disease equation (MDRD-4; re-expressed for an IDMS-traceable assay) with and without adjustment factor for African American ethnicity; and the Chronic Kidney Disease-Epidemiology Collaboration (CKD-EPI) equation with and without adjustment factor for African American ethnicity (formulae are in the appendix [p 1]). Kidney function was defined using the Kidney Disease Improving Global Outcomes criteria:^9^ those with no indicators of kidney damage were defined as no chronic kidney disease; eGFR of less than 60 mL/min per 1·73 m^2^ were defined as having low eGFR; ACR of more than 3 mg/mmol were defined as having albuminuria; and either low eGFR or albuminuria, or both, were defined as having chronic kidney disease. Stages of eGFR were defined according to Kidney Disease: Improving Global Outcomes guidelines for severity.^9,10^ Since this study was cross-sectional, repeat measurements were not done.

Blood pressure was measured three times while participants were seated, with 2-min intervals between measurements. The mean of the last two measurements was used for analysis. Hypertension was defined as mean systolic blood pressure of 140 mm Hg or higher, mean diastolic blood pressure of 90 mm Hg or higher, a history of previously diagnosed hypertension, or current use of antihypertensive medication, irrespective of the blood pressure measurement, or a combination of these.

Diabetes was defined as fasting blood glucose concentration of 7·0 mmol/L or higher, a random glucose concentration measurement of more than 11·1 mmol/L, a history of previously diagnosed diabetes, or current use of antidiabetic medication, irrespective of the glucose measurement, or a combination of these.

Participants also completed a lifestyle and medical history questionnaire. HIV status was by self-report or a positive HIV rapid test. Participants from Kenya and South Africa who tested positive for HIV with voluntary testing were referred to a health-care facility for confirmatory testing and initiation of treatment, as per standard of care in each country. Since HIV prevalence is less than 1% in Ghana and Burkina Faso, participants who had not been tested previously or had tested negative were considered uninfected and not offered further testing.

Self-reported cardiovascular disease was defined as a history of stroke, heart attack, angina, or a transient ischaemic attack.

Information was collected for highest level of education (no formal education, primary, secondary, and tertiary); current smoking (yes or no); and current alcohol consumption (yes or no). Socioeconomic status was estimated using a household assets-based score with classification according to quintiles as implemented by the Demographic and Health Surveillance programme.^6^

### Statistical analysis

We present continuous data as means and SDs and categorical variables as absolute numbers with percentages and 95% CIs. We first tested the difference in eGFR between the sexes for each site using Student’s *t* test, reported as mean (SD), and after which we used general linear models for sex × site model interaction. We standardised age-adjusted prevalence and 95% CIs of indicators of kidney damage (ie, low eGFR, albuminuria, and chronic kidney disease) using the UN World Population for Africa database. For statistical differences between sexes, we used linear models adjusted for age. We also report age-adjusted total prevalences (95% CI) of categorical variables and report means (SD) for continuous variables. We calculated differences between those individuals with no missing data with those with missing data and compared prevalences using Fisher’s test, and compared means using the Wilcoxon test.

We analysed associations between indicators of kidney damage (low eGFR, albuminuria, or chronic kidney disease) and relevant risk factors using relative risk (RR) with logistic regression models. For each pair of risk factors, we defined a covariable set using directed acyclic graphs with a six-step algorithm.^11^ We built the directed acyclic graphs using Bayesian inference methods (for detailed methods see appendix [pp 1–2, 5–6]). Women in Soweto did not have urine samples taken; therefore, participants from Soweto were excluded from the calculation of prevalence and relative risk of albuminuria and chronic kidney disease when calculated for all individuals. Self-reported data for history of cardio vascular disease were not collected for women and for alcohol consumption were not collected for men or women in Soweto.

We did all statistical analyses and figures using R, and in the comparison of prevalence by site we used an adjustment by age and sex using the epitools R-package.

### Role of the funding source

The funders of the study had no role in the study design, data collection, data analyses, data interpretation, or writing of the manuscript. The joint first authors (JAG and J-TB) and senior author (MR) had full access to all the data in the study and the corresponding author (JAG) had final responsibility for the decision to submit for publication.

## Results

Between August, 2013, and August, 2016, 10 702 people were recruited to participate in the AWI-Gen study, of whom 2592 had missing data on key variables relevant to this study, such that 8110 participants had both ACR and eGFR data and an additional 906 women from Soweto had just eGFR values. To address possible bias due to missing data, we calculated the distribution of missing data according to sex and site (appendix p 8–9) and compared key variables between participants with missing data with those with no missing data (excluding women from Soweto). eGFR for participants with no missing data (n=8110) was 98·1 mL/min per 1·73 m^2^ (SD 16·4) compared with 97·9 mL/min per 1·73 m^2^ (SD 14·7) for those with missing data (n=605; p=0·07). Due to the missing ACR data for Soweto women, men and women from Soweto were excluded when assessing prevalence and associated risk factors for all sites combined to minimise bias. Bias due to missing data is therefore unlikely to have a meaningful effect on our results.

Baseline demographic and clinical characteristics for the analysable population (n=8110) with both ACR and eGFR data are shown in table 1 and for the women from Soweto are in the appendix (p 7). Of the analysable population, 4120 (50·8%) were male, with a mean age of 49·9 (SD 5·8). Participants with chronic kidney disease were older, had a higher BMI and waist circumference, and were more likely to have HIV, diabetes, hypertension, and higher levels of education than those with no chronic kidney disease. Total cholesterol, triglycerides, and LDL cholesterol were higher and HDL cholesterol was lower in those with chronic kidney disease than among those with no chronic kidney disease (table 1).

**Figure 1:**
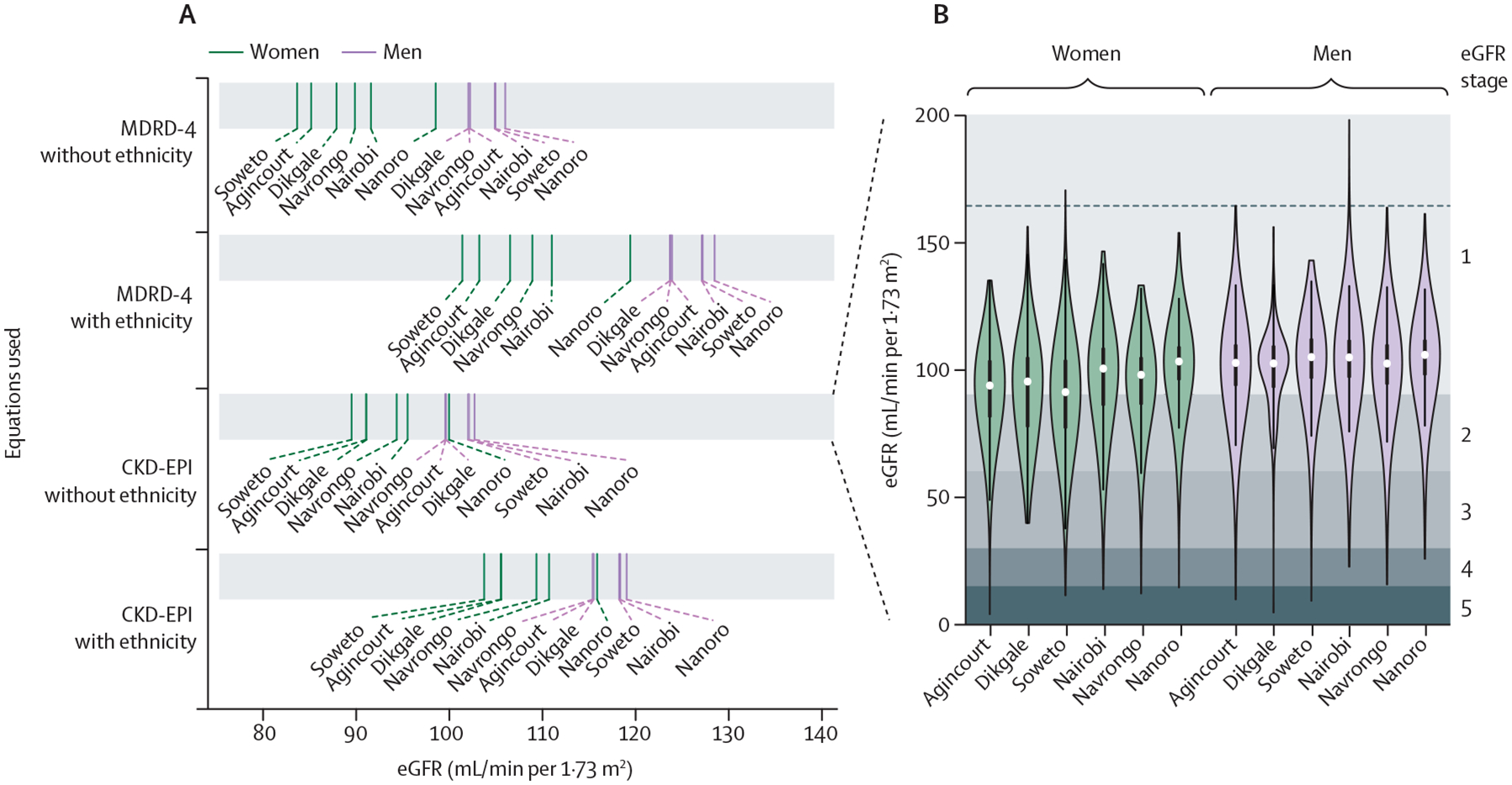
eGFR, by site and sex (A) Comparison of mean eGFR using the MDRD-4 and CKD-EPI equations across AWI-Gen study sites, by sex, with and without inclusion of the African American ethnicity factor; full data are shown in the appendix (p 10). (B) Distribution of eGFR (calculated using CKD-EPI equation without the ethnicity factor), by sex and study site; data shown as median (white dot) and IQR distribution (bold lines), with eGFR stages 1–5 shown by the grey shading. AWI-Gen=Africa Wits-International Network for the Demographic Evaluation of Populations and their Health Partnership for Genomic Studies. CKD-EPI=Chronic Kidney Disease–Epidemiology Collaboration. eGFR=estimated glomerular filtration rate. MDRD-4=4-variable Modification of Diet in Disease.

Women had lower mean eGFR than men at each site, irrespective of the equation used (figure 1; appendix p 10), and a higher prevalence of low eGFR (3·0%, 95% CI 2·6–3·6) than men (1·7% [1·3–2·3]) across all sites (table 2). Of the women from Soweto, 40 (4·4%) of 906 had low eGFR and 866 (95·6%) had normal eGFR appendix (p 7). MDRD-4 equations showed larger differences and higher mean values for men and women than the CKD-EPI equations (figure 1). Inclusion of the African American ethnicity factor resulted in a likely overestimation of eGFR, as has been previously described,^12^ and a higher prevalence of low eGFR (figure 1; appendix pp 10–11). After comparison of the equations, for all subsequent analyses for eGFR we used the CKD-EPI equation without adjustment factor for African American ethnicity.^12^

**Figure 2:**
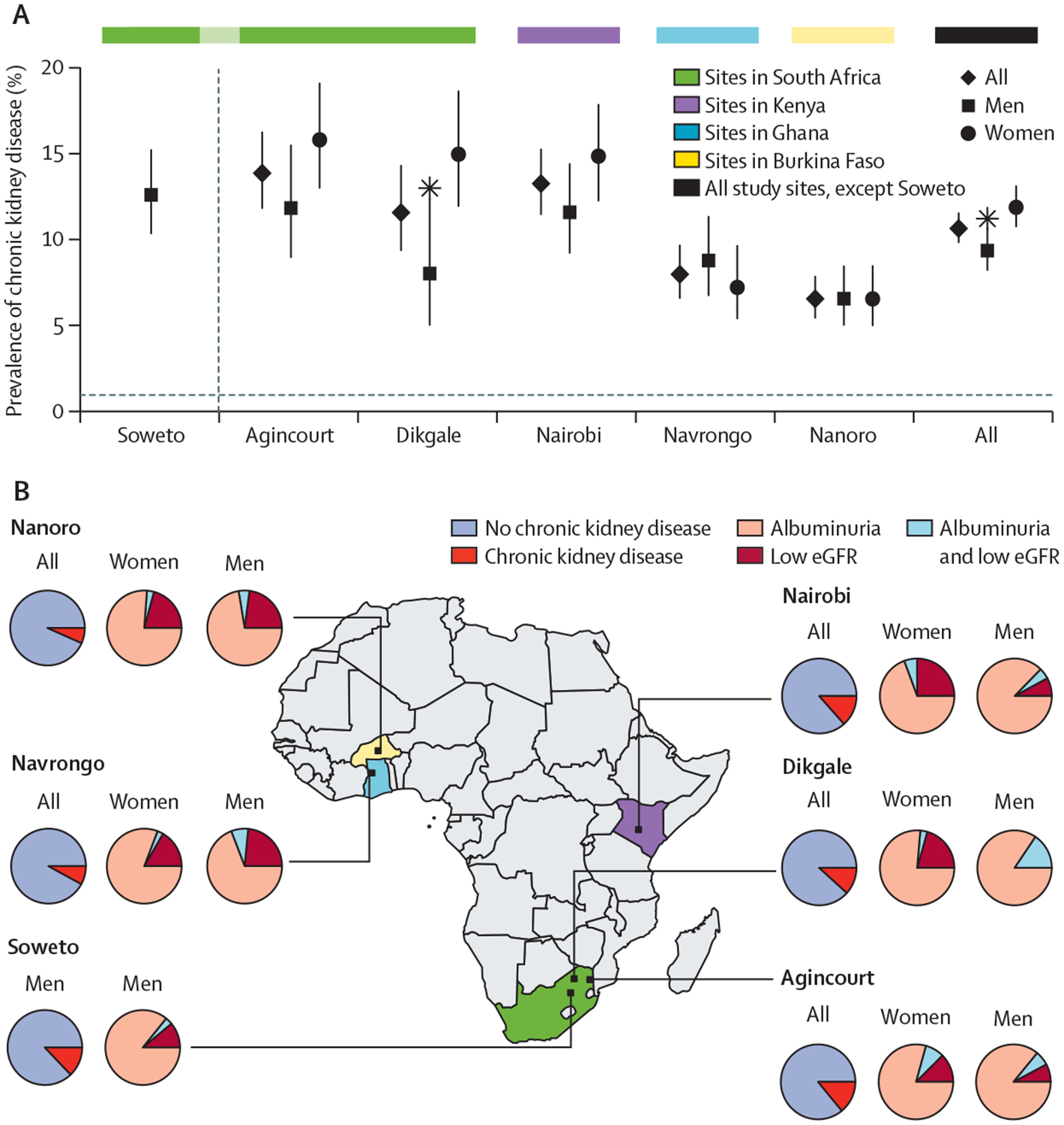
Prevalence of chronic kidney disease in four sub-Saharan African countries (A) Prevalence of chronic kidney disease, by sex and study site, with 95% CIs shown as error bars and significant differences (p<0·05) between men and women, adjusted for age, are shown by stars. (B) Map of Africa showing the locations of the study sites, with the proportion of the population with chronic kidney disease shown in the pie charts. The pie charts labelled women and men show only the individuals with chronic kidney disease from each of the study sites and the relative proportions with low eGFR or albuminuria, or both. eGFR=estimated glomerular filtration rate. Significant differences (p<0·05) between men and women, adjusted for age.

The overall age-adjusted prevalence of chronic kidney disease was 10·7% (95% CI 9·9–11·7), low eGFR was 2·4% (2·1–2·8), and albuminuria was 9·2% (8·4–10·0). Overall prevalence of chronic kidney disease was higher in women (12·0% [10·8–13·2]) than in men (9·5% [8·3–10·8]; p<0·01; table 2); by site, significant sex differences in prevalence were only present in Dikgale (South Africa; figure 2). However, the prevalence of albuminuria was not significantly different between the sexes (p>0·05; table 2). Women had a higher prevalence of low eGFR than did men (3·0% [95% CI 2·6–3·6] *vs* 1·7% [1·3–2·3]; p<0·001).

Age-adjusted prevalence of chronic kidney disease varied by site, ranging from 14·0% (95% CI 11·9–16·4) in Agincourt to 6·6% (5·5–7·9) in Nanoro (figure 2, table 2). Albuminuria contributed more to chronic kidney disease prevalence than did low eGFR (figure 2) and 61 (6·6%) of 928 participants with chronic kidney disease had both albuminuria and low eGFR. 25 (2·7%) participants with chronic kidney disease had self-reported kidney disease (data not shown).

Women in our analysable population had a significantly higher BMI than did men (27·1 kg/m^2^ [SD 7·6] *vs* 22·7 kg/m^2^ [4·6]; p≤0·0001) and a higher prevalence of HIV (17·1% [95% CI 15·7–18·7] *vs* 14·7% [13·2–16·4]; p≤0·0001), hypertension (35·3% [33·5–37·1] *vs* 29·8% [28·0–31·8]; p≤0·0001), and diabetes (6·2% [5·5–7·1] *vs* 4·8% [4·1–5·8]; p≤0·01) than did men (table 2). The prevalence of these comorbidities varied by site and were generally highest in the South African sites and lowest in Nanoro. Characterisation of the risk factors of socioeconomic status and educational attainment are in the appendix (appendix p 13). In Agincourt and Dikgale, more women had higher socioeconomic status than men than at the other sites, and more men had tertiary education at all sites than did women.

Common risk factors for low eGFR, albuminuria, and chronic kidney disease were older age, positive HIV status, diabetes, and hypertension (table 3). Of 768 participants (with complete data for diabetes, hypertension, and HIV) with chronic kidney disease, 495 (63·5%) had one or more of the following risk factors: diabetes mellitus, hypertension, or HIV, and 2268 (36·5%) of 6221 participants with no chronic kidney disease had one or more of these risk factors (appendix p 4).

At all sites, age, diabetes, and hypertension were associated with increased risk for chronic kidney disease (p<0·0001 for analysable population); however, we noted inter-site differences in RR (table 4). For example, the RR for chronic kidney disease in those with diabetes was highest in Nanoro at 2·99 (95% CI 1·59–5·17) and lowest in Agincourt at 1·86 (1·16–2·84). People with hypertension had the highest risk of chronic kidney disease in Soweto (men only: 2·62 [1·67–4·22]) and lowest risk in Agincourt (1·44 [1·06–1·97]). Older age conferred a similar risk across all sites. Risk of chronic kidney disease was increased by positive HIV status in Agincourt and Nairobi, current drinking in Nairobi, and current smoking in Nanoro (table 4). Low socioeconomic status was associated with chronic kidney disease in Nairobi (0·86 [95% CI 0·76–0·96]). High BMI was associated with a slightly increased risk of chronic kidney disease among men in Soweto (1·03 [1·00–1·06]). Risk factors for albuminuria and low eGFR are shown in the appendix (p 14). Diabetes increased risk for albuminuria at all sites except Navrongo sites, ranging from 3·08 (1·49–5·71) in Nanoro to 2·11 (1·30–3·27) in Agincourt. Age was most consistently associated with low eGFR across sites except for Soweto (1·08 [1·02–1·14] in Nanoro to 1·14 [1·07–1·22] in Agincourt).

## Discussion

To our knowledge, this is the first large-scale population-based study from sub-Saharan Africa to investigate kidney damage and associated risk factors in adults from South Africa and countries in west and east Africa. Using low eGFR and albuminuria to define chronic kidney disease, we showed a regional prevalence of chronic kidney disease of 10·7%, with the largest relative contribution from albuminuria. We showed differences in chronic kidney disease prevalence between women (12·0%) and men (9·5%) that were primarily due to differences in low eGFR rather than albuminuria. Notably, prevalence of chronic kidney disease was substantially different between sites and regions, with the highest prevalence in the south and the lowest prevalence in the west. Associated risk factors for chronic kidney disease across all sites, although to varying degrees, included older age, positive HIV status, diabetes, and hypertension. In a systematic review and meta-analysis by Stanifer and colleagues,^4^ the estimated prevalence of chronic kidney disease in Africa was 13·9% and although our estimated prevalence is lower, it is higher than published data from smaller studies done in Kinshasa, Democratic Republic of the Congo (7·8%), and Morocco (5·1%).^13,14^ Regional differences in the prevalence of chronic kidney disease in sub-Saharan Africa are in part due to the different methods used by investigators—eg, study design, sampling, definitions of chronic kidney disease, laboratory methods for creatinine measurement, and lack of a validated measure for eGFR in Africans. In this study, we used population-based sampling frames, a single laboratory for all serum and urine testing, applied the same definitions for kidney damage, and used the same eGFR equation across all sites, all of which strengthen the likelihood that our observed inter-site differences in the prevalence of chronic kidney disease are likely to be more accurate than previous studies.

The prevalence of low eGFR, albuminuria, and chronic kidney disease and associated risk factors (eg, BMI in women and hypertension in both sexes) were higher in the eastern (Nairobi) and southern sites (Agincourt, Dikgale, and Soweto) than in the western sites (Nanoro and Navrongo). This pattern might be explained by varying stages of rapid health and sociodemographic transition, possibly led by the south and east of the continent, as supported by mortality trends.^15^ In South Africa, Agincourt has high mortality from HIV and tuberculosis, with increasing mortality due to non-communicable diseases such as stroke, while in Nanoro mortality due to malaria is decreasing and the shift to an increase in non-communicable diseases has not yet occurred.^15^ Socioeconomic status and the accompanying differences in lifestyle also vary substantially across sub-Saharan Africa. For example, South Africa has the highest gross national income according to the World Bank compared with the other African countries in this study.^16^ Data from the RODAM study in Ghana^17^ and a study in Tanzania,^18^ showed a higher prevalence of chronic kidney disease in people who lived in urban areas than in those who lived in rural areas, which they attributed to lower prevalence of conventional risk factors in those living in rural areas. However, in our study, we found no major difference in prevalence of chronic kidney disease between men in Soweto, an urban metropole, and the populations of Agincourt and Dikgale, which are semi-rural areas. Agincourt, although semi-rural, has had an overall increase in wealth over the past 25 years, which might explain the attenuated differences we have observed between urban and rural sites in South Africa.^19^

In the USA, the higher prevalence of chronic kidney disease and progression to end-stage kidney disease in African Americans than in other population groups might partly be attributable to the presence of chronic kidney disease risk variants of apolipoprotein L1 (*APOL1*). These variants confer a survival advantage against trypanosomiasis but increase glomerular scarring in response to conditions such as hypertension, diabetes, and HIV.^20^ The relationship between *APOL1* risk variants and kidney disease in Africans is complex. The frequency of *APOL1* risk variants tends to be high in Ghana and Nigeria^21^ and our data show a lower prevalence of chronic kidney disease in Ghana and Burkina Faso with a higher prevalence in South Africa, where *APOL1* risk variants are less common. This observation suggests that *APOL1* variants are neither sufficient nor essential for chronic kidney disease in Africans and that other factors (eg, JC virus is protective; positive HIV status increases risk) are important in gene–environment interactions.

Presence of hypertension doubled the risk of chronic kidney disease at most sites and diabetes was associated with a two-to-three times increased relative risk of chronic kidney disease. Perhaps even more notably, more than a third of participants with chronic kidney disease did not have the well described risk factors associated with chronic kidney disease—ie, HIV, diabetes, or hypertension—and this finding was congruent with other epidemiological studies from sub-Saharan Africa,^4,18^ suggesting that unmea sured risk factors for chronic kidney disease exist in our participants that we did not investigate. A higher proportion of people who consumed alcohol resided in Navrongo and Nanoro than in the other sites and the lower prevalence of chronic kidney disease at these sites might be related to a higher prevalence of chronic liver disease and consequent lower serum creatinine levels. Differences in disease awareness, risk factors, and treatment might contribute to the varying prevalences in each region. For example, in a study of hypertension prevalence and awareness in the AWI-Gen cohort, 576 (39·4%) of 1503 men were aware of their disease compared with 1091 (53·8%) of 2040 women.^22^ The same had been shown to be true of dia betes in our study cohort (Crowther NJ, unpublished).

The reasons for the increased prevalence of low eGFR and chronic kidney disease among women are multi factorial and likely include a higher prevalence of associated risk factors (hypertension, diabetes, HIV, and obesity), gender-based differences that influence access to care, and biological factors.^23,24^ Previous studies have indicated that chronic kidney disease in women increases risk of pregnancy-related complications like pre-eclampsia, premature birth, and small for gestational age or low birthweight babies, and consequently fewer nephrons in their offspring, making this area compelling for further investigation.^23^

An important limitation of the diagnosis of chronic kidney disease in sub-Saharan Africa is lack of a validated measure of eGFR in local populations. Our findings show that, depending on the equation used, calculated prevalence can vary substantially. The overestimation of eGFR with the use of the African American ethnicity factor^12,25^ is possibly because of differences in muscle bulk and body composition between Africans and African Americans.^12^ Another reason might be that the correction factor for body surface area is inappropriate for women. Men tend to have a greater body surface area, which is the main predictor of kidney size;^26^ therefore, correcting eGFR by a constant that is not sex specific might not achieve reliable measurements. The diagnosis of kidney disease and diabetes is dependent on accurate laboratory measurements and, although laboratory services across Africa have been improved in a bid to enhance HIV care,^27^ biomarkers for non-communicable diseases have yet to receive much attention.

Because this study was cross sectional, we did not show chronicity and might have overestimated chronic kidney disease. Repeat creatinine measurements in a study from Morocco showed that 32% of patients with stage 3a chronic kidney disease when initially tested did not have low eGFR on retesting.^14^ The use of a single cutoff to define low eGFR might lead to overdiagnosis in older participants and underdiagnoses in younger participants.^14^ We did not test urine for haematuria or for urinary sediment and thus might have underestimated the prevalence of chronic kidney disease due to glomerulonephritis. Additionally, we did not collect data on heart failure, which could result from hypertension and contribute to cardiorenal syndrome, and we have not yet done genetic studies and genetic variation might have an important role in susceptibility to chronic kidney disease.^20^ Hypertension might also be a consequence of chronic kidney disease; however, this reverse association was not assessed in our study. Use of angiotensin-converting-enzyme inhibitors could be the cause of both low eGFR and albuminuria for a few participants. We do not have data on specific medications that participants were receiving; however, published data from AWI-Gen has shown that hypertension awareness, treatment, and control are poor in this cohort^22^ and therefore the effect of any angiotensin-converting-enzyme inhibitors is likely to be low. The participant sampling approaches varied slightly between study sites and might have had a small effect on the reported prevalence of chronic kidney disease.

Our study was sufficiently large to explore the more common risk factors for chronic kidney disease and differences in prevalence across regions, using rigorous standardised protocols across all study sites. All assays were done in the same laboratory with strict quality control procedures to ensure consistency of results using a combination of eGFR and urinary ACR to define chronic kidney disease. This is, to our knowledge, the first study that included four countries from sub-Saharan Africa at varying stages of development with a mix of urban and rural populations, providing an important opportunity to explore the effect of multiple risk factors on the burden of chronic kidney disease in different settings.

Our data show that chronic kidney disease is an important public health problem in sub-Saharan Africa and risk is strongly associated with traditional risk factors such as increased age, diabetes, and hypertension.^4,13,28,29^ With evidence of a rapid epidemiological health transition, economic growth, urbanisation, and an increasing older population in many regions, more Africans are at an increased risk of chronic kidney disease.^30,31^ Our findings highlight the need for strategic and appropriate public health policy and health systems interventions that support integrated screening and prevention strategies for chronic kidney disease in existing programmes that manage infectious diseases and non-communicable diseases. Implementation of the WHO best buys interventions, such as taxes on tobacco and alcohol and reducing salt intake, will help to reduce the risk of non-communicable diseases. South Africa has implemented a sugar tax in a bid to curb the epidemic of obesity and the salt content of widely consumed products, such as bread, has been reduced.

Larger-scale epidemiological studies are needed to examine many potential but currently unmeasured urban risk factors including contaminated water supplies, occupational exposures, use of over-the-counter analgesics, traditional medicines, and infectious diseases like tuberculosis. Genetic differences and intrauterine fetal exposure have important roles in susceptibility to chronic kidney disease and careful longitudinal studies of the interaction between genetic risk factors and fetal, childhood, and adult environmental exposures, including nutrition, will be crucial in understanding the aetiology of chronic kidney disease in Africa.

## Supplementary Material

Supplementary material

## Figures and Tables

**Table 1:** Demographic and clinical characteristics of AWI-Gen participants

	No chronic kidney disease (n=7182)	Low eGFR (n=218)	Albuminuria (n=771)	Chronic kidney disease (n=928)	Total population (n=8110)*
Sex					
Overall					
Men	3677 (51·2%)	90 (41·3%)	379 (49·2%)	439 (47·3%)	4120 (50·8%)
Women	3505 (48·8%)	128 (58·7%)	392 (50·8%)	489 (527%)	3990 (49·2%)
Agincourt					
Men	436/1077 (40·5%)	12/44 (27·3%)	65/178 (36·5%)	71/203 (35·0%)	507/1280 (39·6%)
Women	641/1077 (59·5%)	32/44 (72·7%)	113/178 (63·5%)	132/203 (65·0%)	773/1280 (60·4%)
Dikgale					
Men	254/751 (33·8%)	5/29 (17·2%)	25/103 (24·3%)	26/123 (21·1%)	280/874 (32·0%)
Women	497/751 (66·2%)	24/29 (82·8%)	78/103 (757%)	97/123 (78·9%)	594/874 (68·0%)
Nairobi					
Men	636/1327 (47·9%)	11/46 (23·9%)	79/170 (46·5%)	85/203 (41·9%)	721/1530 (47·1%)
Women	691/1327 (52·1%)	35/46 (76·1%)	91/170 (53·5%)	118/203 (58·1%)	809/1530 (52·9%)
Nanoro					
Men	872/1738 (50·2%)	21/37 (56·8%)	52/103 (50·5%)	68/133 (51·1%)	941/1871 (50·3%)
Women	866/1738 (49·8%)	16/37 (43·2%)	51/103 (49·5%)	65/133 (48·9%)	930/1871 (49·7%)
Navrongo					
Men	718/1528 (47·0%)	25/46 (54·3%)	55/114 (48·2%)	74/151 (49·0%)	793/1679 (47·2%)
Women	810/1528 (53·0%)	21/46 (45·7%)	59/114 (51·8%)	77/151 (51·0%)	886/1679 (52·8%)
Soweto†					
Men	761/761 (100%)	16/16 (100%)	103/103 (100%)	115/115 (100%)	876/876 (100%)
Women	NA	NA	NA	NA	NA
Age, years	49·8 (5·8)	52·9 (5·4)	50·9 (5·8)	51·2 (5·8)	49·9 (5·8)
Body·mass index					
n	7174	218	770	927	8101
Mean, kg/m^2^	24·0 (6·0)	25·1 (6·9)	25·1 (6·7)	25·1 (6·8)	24·2 (6·11)
Waist circumference					
n	7169	218	769	926	8095
Mean, cm	83·7 (13·9)	86·6 (15·3)	87·5 (15·9)	87·3 (15·9)	84·1 (14·2)
Education					
No formal education	2968/7167 (41·4%)	83/218 (38·1%)	239/767 (31·2%)	299/924 (32·4%)	3267/8091 (40·4%)
Primary	1953/7167 (27·2%)	71/218 (32·6%)	272/767 (35·5%)	323/924 (35·0%)	2276/8091 (28·1%)
Secondary	1978/7167 (27·6%)	55/218 (25·2%)	228/767 (29·7%)	268/924 (29·0%)	2246/8091 (27·8%)
Tertiary	268/7167 (3·7%)	9/218 (4·1%)	28/767 (3·7%)	34/924 (3·7%)	302/8091 (3·7%)
Socioeconomic quintile					
Quintile 1	1001/7175 (14·0%)	24/218 (11·0%)	103/770 (13·4%)	119/927 (12·8%)	1120/8102 (13·8%)
Quintile 2	1415/7175 (19·7%)	36/218 (16·5%)	125/770 (16·2%)	152/927 (16·4%)	1567/8102 (19·3%)
Quintile 3	1248/7175 (17·4%)	47/218 (21·6%)	141/770 (18·3%)	178/927 (19·2%)	1426/8102 (17·6%)
Quintile 4	1557/7175 (21·7%)	42/218 (19·3%)	196/770 (25·5%)	226/927 (24·4%)	1783/8102 (22·0%)
Quintile 5	1954/7175 (27·2%)	69/218 (31·7%)	205/770 (26·6%)	252/927 (27·2%)	2206/8102 (27·2%)
HIV positive	805/6996 (11·5%)	40/211 (19·0%)	173/738 (23·4%)	195/889 (21·9%)	970/7885 (12·3%)
Current alcohol consumption	2749/6409 (42·9%)	74/202 (36·6%)	256/667 (38·4%)	311/812 (38·3%)	2722/7221 (37·7%)
Current smoker	1298/7171 (18·1%)	37/217 (17·1%)	153/768 (19·9%)	181/924 (19·6%)	1473/8095 (18·2%)
History of cardiovascular disease	230/7174 (3·2%)	9/218 (4·1%)	33/770 (4·3%)	37/927 (4·0%)	267/8101 (3·3%)
Hypertension	2097/7182 (29·2%)	104/218 (47·7%)	418/771 (54·2%)	486/928 (52·4%)	2587/8110 (31·9%)
Diabetes	306/7124 (4·3%)	21/214 (9·8%)	97/760 (12·8%)	109/915 (11·9%)	410/8039 (5·1%)
Triglycerides					
n	7182	218	771	928	8110
Mean, mmol/L	0·9 (0·5)	1·3 (0·8)	1·0 (0·7)	1·0 (0·7)	0·9 (0·6)
LDL cholesterol					
n	7111	215	765	920	8031
Mean, mmol/L	2·2 (0·9)	2·8 (1·1)	2·3 (0·9)	2·4 (1·0)	2·2 (0·9)
HDL cholesterol					
n	7182	218	771	928	8110
Mean, mmol/L	1·2 (0·4)	1·18 (0·35)	1·22 (0·48)	1·21 (0·46)	1·18 (0·41)
Total cholesterol					
n	7182	218	771	928	8110
Mean, mmol/L	3·8 (1·1)	4·5 (1·4)	4·0 (1·2)	4·1 (1·2)	3·8 (1·1)
Serum creatinine concentration					
n	7182	218	771	928	8110
Mean, pmol/L	67·3 (14·0)	143·6 (101·9)	79·1 (61·7)	86·6 (60·2)	69·5 (25·0)
eGFR					
n	7182	218	771	928	8110
Mean, mL/min per 1·73m^2^‡	997 (14·1)	48·2 (11·9)	92·5 (21·6)	85·4 (25·4)	98·1 (16·4)
ACR					
N	7182	218	771	928	8110
Mean, mg/mmol	0·3 (0·6)	6·3 (13·7)	11·9 (12·7)	10·0 (12·4)	1·4 (5·2)

Data are n (%), n/N (%), n, or mean (SD). AWI-Gen=Africa Wits-International Network for the Demographic Evaluation of Populations and their Health Partnership for Genomic Studies. eGFR=estimated glomerular filtration rate. ACR=albumin to creatinine ratio.

* 61 individuals have both low eGFR and albuminuria, hence total population does not equate to total of columns.

† Women in Soweto are not included because they did not have urine samples taken; baseline data for these women are in the appendix (p 6).

‡ eGFR calculated using Chronic Kidney Disease–Epidemiology Collaboration equation without African American ethnicity factor.

**Table 2:** Characterisation of age-adjusted prevalence of indicators of kidney disease and characterisation of risk factors, by site

	Indicators of kidney disease			Characterisation of risk factors							
	Low eGFR	Albuminuria	Chronic kidney disease	Age, years	BMI, kg/m^2^	HIV	Current smoker	Hypertension	Diabetes	Current alcohol consumption	History of cardiovascular disease
**All sites**											
All	2·4% (2·1–2·8)	9·2% (8·4–10·0)*	10·7% (9·9–11·7)*	49·8 (5·8)	25·1 (6·8)	15·9% (14·9–17·1)	19·4% (18·2–20·6)	32·6% (31·3–34)	5·6% (5·0–6·2)	41·4% (39·7–43·1)*	3·5% (3·0–4·1)*
Women	3·0% (2·6–3·6)†	9·9% (8·8–11·0)*	12·0% (10·8–13·2)‡*	49·9 (5·8)	27·1 (7·6)§	17·1% (15·7–18·7)§	2·3% (1·8–2·8)§	35·3% (33·5–37·1)‡	6·2% (5·5–7·1)*	27·6% (25·9–29·5)§*	4·0% (3·3–4·8)‡*
Men	1·7% (1·3–2·3)	8·4% (7·3–9·7)*	9·5% (8·3–10·8)*	49·7 (5·9)	22·7 (4·6)	14·7% (13·2–16·4)	37·3% (35–39·7)	29·8% (28·0–31·8)	4·8% (4·1–5·8)	55·7% (52·7–58·9)*	3·0% (2·3–4·0)*
**Agincourt**											
All	2·4% (1·7–3·4)	12·7% (10·7–15·1)	14·0% (11·9–16·4)	50·7 (5·8)	27·2 (6·6)	36·9% (33·2–40·9)	12·7% (10·4–15·4)	45·6% (41·8–49·7)	5·0% (3·8–6·6)	22·5% (19·4–25·9)	3·9% (2·9–5·4)
Women	3·1% (2·0–4·7)	14·0% (11·3–17·2)	15·8% (12·9–19·1)	50·8 (5·8)	29·4 (6·6)§	37·3% (32·7–42·4)	0·4% (0·1–1·3)§	52·5% (47·3–58·2)§	4·7% (3·3–6·7)	5·3% (3·7–7·5)§	4·6% (3·1–6·6)
Men	1·6% (0·8–3·2)	11·4% (8·5–15)	12·2% (9·3–15·9)	50·7 (5·9)	24·0 (5·2)	36·4% (30·7–43·0)	25·7% (21–31·2)	38·4% (32·9–44·6)	5·2% (3·4–7·9)	40·4% (34·5–47·1)	3·2% (1·8–5·5)
**Dikgale**											
All	2·3% (1·5–3·8)	10·2% (8·1–12·9)	11·7% (9·5–14·5)	50·3 (6·0)	28·3 (8·4)	21·9% (18·5–25·9)	32·5% (27·8–37·9)	36·7% (32·5–41·3)	7·4% (5·5–9·7)	37·3% (32·4–42·8)	5·9% (4·3–8·1)
Women	3·3% (2·1–5·3)	12·3% (9·5–15·7)	15·1% (12·0–18·8)‡	50·4 (5·9)	31·4 (81)§	24·2% (19·9–29·1)	3·0% (1·7–5·0)§	44·1% (38·7–50·2)§	8·1% (5·9–10·9)	12·7% (9·8–16·3)§	6·0% (4·2–8·6)
Men	1·3% (0·3–3·8)	8·1% (5·1–12·6)	8·1% (5·1–12·6)	50·0 (6·1)	21·7 (4·0)	19·6% (14·3–26·3)	63·4% (53·9–74·3)	28·8% (22·8–36·2)	6·6% (3·8–10·8)	63% (53·5–73·9)	5·7% (3·2–9·6)
**Soweto**											
All*	3·1% (2·3–4·1)	‥	‥	49·2 (5·8)	29·1 (7·7)	22·8% (19·8–26·2)	28·9% (26·4–31·6)	52% (48·7–55·5)	8·9% (7·6–10·4)	‥	‥
Women*	4·4% (3·2–6·1)‡	‥	‥	49·1 (5·6)	33·2 (7·2)§	23·7 (18·7–29·5)	5·2% (3·8–7·0)§	53·7% (49·0–58·8)	11·4% (9·3–13·9)†	‥	‥
Men	1·7% (0·9–2·9)	11·6% (9·4–14·1)	12·9% (10·6–15·5)	49·3 (6·0)	24·9 (5·7)	21·9% (18·7–25·5)	53·7% (48·8–59·0)	50·2% (45·6–55·2)	6·3% (4·8–8·2)	‥	3·3% (2·2–4·7)
**Nairobi**											
All	3·0% (2·1–4·0)	11·2% (9·6–13·1)	13·4% (11·6–15·5)	48·4 (5·4)	25·5 (5·8)	12·7% (10·9–14·8)	13% (11·2–15·0)	25·6% (23·0–28·3)	7·1% (5·8–8·7)	19·1% (16·9–21·5)	4·0% (3·0–5·2)
Women	4·4% (3·1–6·3)‡	11·7% (9·4–14·5)	15·2% (12·5–18·3)	48·1 (5·3)¶	27·8 (6·2)§	16·7% (13·9–20·0)§	2·5% (1·6–4·0)§	29·7% (25·9–33·9)†	9·9% (7·8–12·6)§	6·0% (4·4–8·1)§	5·1% (3·6–7·0¶
Men	1·4% (0·7–2·6)	10·7% (8·4–13·5)	11·6% (9·2–14·4)	48·7 (5·5)	22·8 (4·0)	8·6% (6·4–11·3)	24·0% (20·5–27·9)	21·3% (18·0–25·0)	4·1% (2·8–6·0)	32·7% (28·6–37·3)	2·9% (1·7–4·5)
**Navrongo**											
All	2·0% (1·4–2·9)	6·4% (5·1–8·0)	8·0% (6·6–9·7)	51·0 (5·8)	21·7 (3·7)	0·8% (0·4–1·6)	21·5% (19·1–24·2)	20·8% (18·4–23·4)	1·6% (1·0–2·6)	64·7% (60·4–69·3)	2·4% (1·7–3·5)
Women	1·3% (0·8–2·6)	6·1% (4·4–8·5)	7·3% (5·5–9·7)	51·5 (5·7)†	22·3 (3·9)§	0·6% (0·2–2·0)	2·4% (1·4–4·2)§	20·8% (17·6–24·7)	1·6% (0·7–3·1)	53·9% (48·4–60·1)§	2·6% (1·5–4·4)
Men	2·6% (1·6–4·3)	6·7% (4·9–9·0)	8·7% (6·7–11·3)	50·5 (5·8)	20·9 (3·3)	1·0% (0·4–2·2)	41·4% (36·8–46·7)	20·7% (17·5–24·4)	1·6% (0·8–3·1)	76·0% (69·6–83·0)	2·3% (1·3–3·9)
**Nanoro**											
All	1·7% (1·1–2·4)	5·2% (4·2–6·4)	6·6% (5·5–7·9)	49·8 (5·8)	20·9 (3·4)	0·4% (0·2–0·9)	7·5% (6·3–9·0)	15·1% (13·3–17·0)	3·4% (2·6–4·4)	63·2% (59·5–67·1)	1·4% (0·9–2·1)
Women	1·5% (0·9–2·6)	5·2% (3·8–7·0)	6·6% (5·0–8·5)	49·8 (5·7)	20·2 (3·1)§	0·3% (0·0–1·1)	0%	10·8% (8·8–13·2)§	1·8% (1–2·9)†	60·2% (55·1–65·6)‡	1·7% (0·9–2·9)
Men	1·8% (1·0–3·0)	5·2% (3·8–7·0)	6·7% (5·1–8·6)	49·8 (6·0)	21·6 (3·6)	0·6% (0·2–1·4)	15·4% (12·8–18·3)	19·6% (16·8–22·7)	5·1% (3·7–6·9)	66·4% (61·0–72·1)	1·1% (0·6–2·1)

Data are mean (SD) or proportion with 95% CI in parentheses. For statistical differences between sexes for all variables, we used linear models adjusted for age.

* Women from Soweto were excluded from analyses.

† p≤0·001.

‡ p≤0·01.

§ p≤0·0001

¶ p≤0·05.

**Table 3:** Risk factors associated with low eGFR, albuminuria, and chronic kidney disease

	Low eGFR		Albuminuria*		Chronic kidney disease*	
	Relative risk	p value	Relative risk	p value	Relative risk	p value
Male sex	0·76 (0·57–1·01)	0·061	0·89 (0·76–1·05)	0·16	0·85 (0·73–0·98)	0·018
Age	1·10 (1·07–1·12)	<0·0001	1·03 (1·01–1·04)	0·0001	1·04 (1·03–1·05)	<0·0001
Body-mass index	1·02 (0·99–1·04)	0·15	1·00 (0·99–1·02)	0·65	1·01 (1·00–1·02)	0·19
Diabetes	1·73 (1·08–2·63)	0·012	2·37 (1·84–3·01)	<0·0001	2·22 (1·76–2·78)	<0·0001
Highest level of education	1·13 (0·93–1·37)	0·21	1·00 (0·89–1·12)	0·97	1·03 (0·93–1·14)	0·56
HIV positive	1·46 (1·00–2·10)	0·051	1·97 (1·60–2·42)	<0·0001	1·65 (1·36–1·99)	<0·0001
Hypertension	1·63 (1·22–2·17)	0·00088	2·07 (1·75–2·44)	<0·0001	1·97 (1·68–2·30)	<0·0001
Socioeconomic status	1·01 (0·92–1·12)	0·82	1·00 (0·95–1·06)	0·96	1·00 (0·95–1·05)	0·97
Current smoker	1·05 (0·72–1·50)	0·79	1·10 (0·84–1·42)	0·49	1·16 (0·91–1·47)	0·23
Current alcohol consumption*	0·88 (0·63–1·21)	0·43	1·21 (1·00–1·47)	0·051	1·16 (0·98–1·38)	0·092
History of cardiovascular disease*	1·08 (0·49–2·06)	0·83	1·11 (0·74–1·59)	0·59	1·06 (0·73–1·49)	0·75

Data are relative risk, with 95% CI in parentheses, and p values for various risk factors, with cofactors defined with directed acyclic graphs and six-step algorithms are shown in the appendix (p 14). 6941 people were included in the analyses of relative risk of albuminuria and chronic kidney disease and 8129 were included in the analysis for relative risk of low eGFR 8129. eGFR=estimated glomerular filtration rate.

* All participants from Soweto were excluded from the calculation of albuminuria and chronic kidney disease since the women did not have urine samples taken and did not have data collected on history of heart disease and neither men nor women had data on alcohol consumption.

**Table 4:** Risk factors associated with chronic kidney disease, by study site

	Agincourt (n=126l)	Dikgale (n=844)	Nairobi (n=1356)	Nanoro (n=l850)	Navrongo (n=l630)	Soweto (n=825)*
Male sex	0·81 (0·60–1·08)	0·61 (0·38–0·93)†	0·71 (0·52–0·97)†	1·04 (0·74–1·46)	1·09 (0·79–1·52)	‥
Age	1·04 (1·01–1·06)‡	1·04 (1·01–1·08)‡	1·04 (1·01–1·07)‡	1·04 (1·01–1·07)‡	1·04 (1·01–1·07)†	1·04 (1·01–1·07)†
Body-mass index	1·01 (0·99–1·04)	1·00 (0·97–1·03)	1·00 (0·97–1·03)	0·98 (0·92–1·03)	0·99 (0·95–1·04)	1·03 (1·00–1·06)†
Diabetes	1·86 (1·16–2·84)‡	2·28 (1·39–3·58)§	2·29 (1·46–3·45)§	2·99 (1·59–5·17)§	1·95 (0·59–4·68)	2·62 (1·51–4·31)§
Highest level of education	1·01 (0·85–1·19)	1·11 (0·85–1·46)	0·96 (0·75–1·24)	0·99 (0·69–1·35)	1·10 (0·87–1·36)	0·75 (0·54–1·06)
HIV positive	1·41 (1·06–1·86)†	1·35 (0·88–2·01)	2·39 (1·68–3·33)¶	‥||	‥||	1·25 (0·79–1·90)
Hypertension	1·44 (1·06–1·97)†	1·77 (1·19–2·65)‡	2·31 (1·68–3·16)¶	2·10 (1·43–3·03)§	2·30 (1·64–3·20)¶	2·62 (1·67–4·22)
Socioeconomic status	1·00 (0·90–1·10)	1·05 (0·92–1·19)	0·86 (0·76–0·96)‡	1·10 (0·97–1·25)	1·04 (0·92–1·17)	0·99 (0·83–1·18)
Current smoker	0·79 (0·44–1·38)	0·33 (0·15–0·71)‡	1·46 (0·89–2·32)	2·13 (1·21–3·59)‡	1·37 (0·87–2·14)	1·03 (0·70–1·52)
Current alcohol consumption	1·37 (0·92–2·00)	1·02 (0·61–1·66)	1·62 (1·09–2·36)†	1·12 (0·79–1·63)	1·01 (0·71–1·44)	‥
History of cardiovascular disease	0·76 (0·32–1·49)	1·03 (0·46–1·98)	1·29 (0·61–2·38)	1·31 (0·32–3·48)	1·35 (0·48–2·96)	‥

Data are relative risk, with 95% CIs in parentheses. Relative risk for various effectors, with cofactors defined with directed acyclic graphs and six-step algorithms shown in the appendix (pp 13, 15). p values are derived from generalised linear model comparisons for each risk factor, for categorical variables this comparison was to the appropriate reference group—eg, diabetic *vs* non-diabetic—whereas for continous variables, such as age and body-mass index, an increase in risk is donoted by a 1 unit change in the variable.

* Soweto participants did not have sufficient data on history of cardiovascular disease and alcohol consumption.

† p≤0·05.

‡ p≤0·01.

§ p≤0·001.

¶ p≤0·0001.

|| Because HIV prevalence is less than 1% in Ghana and Burkina Faso, participants who had not been tested previously or had tested negative, were considered uninfected, and not offered further testing; therefore no data were available.

## Research in context

### Evidence before this study

We searched PubMed for reports published between Jan 31, 2008, and Dec 31, 2017, on chronic kidney disease in Africa using the terms “chronic kidney disease”, “renal insufficiency”, and “Africa epidemiology”. African studies were generally small and reported data at national and community levels with cross-sectional prevalence surveys for chronic kidney disease included in several systematic reviews and meta-analyses, but few population-level data were available for studies across more than one African country.

### Added value of this study

We used harmonised data to assess the prevalence of chronic kidney disease in four sub-Saharan African countries using both albuminuria and estimated glomerular filtration rate. Chronic kidney disease is more prevalent in women and shows an increasing prevalence with age. Regionally, chronic kidney disease is more prevalent in South Africa and Kenya than in Ghana and Burkina Faso. Individuals with hypertension, diabetes, and positive HIV status were at highest risk; however, a substantial proportion of people with the disease did not have traditional risk factors.

### Implications of all the available evidence

Chronic kidney disease is widespread in Africa and prevalence is higher in countries with more advanced economies. Access to treatment for chronic kidney disease is restricted and action is needed to reduce the burden of risk factors and determine the impact of non-traditional risk factors on chronic kidney disease prevalence. A multisectoral approach is essential, involving government, industry, health-care providers, and the community to develop evidence-informed context-appropriate strategies for prevention and treatment of chronic kidney disease.
