# Supplementary material for: Kidney damage and associated risk factors in rural and urban sub-Saharan Africa (AWI-Gen): a cross-sectional population study
Source: Lancet Glob Health. Author manuscript; Available in PMC 2020 Feb 21. (PMC7033368; doi:10.1016/S2214-109X(19)30443-7)
Supplement: Supplementary material [file NIHMS1558789-supplement-Supplementary_material.pdf]

THE LANCET
Global Health

Supplementary appendix

This appendix formed part of the original submission and has been peer reviewed. We post it as supplied by the authors.

Supplement to: George JA, Brandenburg J-T, Fabian J, et al. Kidney damage and associated risk factors in rural and urban sub-Saharan Africa (AWI-Gen): a cross-sectional population study. *Lancet Glob Health* 2019; **7:** e1632–43.

Appendix

eGFR Equations

MDRD-4: Re-expressed* 4-variable Modification of Diet in Renal Disease equation
eGFR ($mL/min/1.73m^2$) = 175 x $(S\text{-}Cr)^{-1.154}$ x age $(years)^{-0.203}$ x (0.742 if female) x (1.1212 if African American)
*re-expressed using an IDMS-traceable assay to a standard reference material

CKD-EPI: Chronic Kidney Disease Epidemiology Collaboration equation
eGFR ($mL/min/1.73m^2$) = 141 × min (S-Cr/κ, 1) α × max (S-Cr/κ, $1)^{-1.209}$ × 0.993^{age} × 1.018 [if female] × 1.159 [if black]
S-Cr is serum creatinine in μmol/L,
κ is 61.9 for females and 79.6 for males,
α is -0.329 for females and -0.411 for males,
min indicates the minimum of S-Cr/κ or 1 and max indicates the maximum of S-Cr/κ or 1

Choice of variables to compute risk factors with *6-step DAG* algorithm

Usually, in epidemiology, researchers present a specific outcome (i.e. CKD, low eGFR or albuminuria) and one or more exposures/confounders (i.e. sex, age, site, SES) for a single logistic regression or generalized linear model. These approaches may affect and bias estimates of exposure/effector on outcome[1]. To avoid bias, we defined risk factors with generalized linear models for each effector/outcome pair with a specific set of confounders. We selected the best set of confounders for each pair with the 6-step algorithm of Shrier and Platt[2] as applied to Directed Acyclic Graphs (DAG)[3,4].

Building DAG

DAGs have been used in epidemiology to represent causal relationships among variables, and they have been used extensively to determine which variables are necessary to condition on in order to control for confounding. DAGs have properties to indicate the direction of the effect and do not generate internal cycles. For each outcome, we built a specific DAG with other variables (see list in Box below) using the relationship observed between variables in the dataset. We used a similar procedure of "Bayesian network and inference" as used in reference 4, with the Grow-Shrink algorithm[5] in association with the mutual information test defined for hybrid Bayesian networks implemented in R package bnlearn[6]. To determine the direction of the link, we defined some impossible causal relationships between variables in the data (as shown below in the box with columns A and B). DAG diagrams are presented in Supplementary Figure 3.

Box: Impossible causal relationships between A=> B to build DAG with bnlearn library

(Abbreviations: DM - Diabetes, HTP - hypertension, Drk - Current drinker, Smk - Current Smoker)

A	B
Age	Sex,Site
BMI	Sex,Site,Age,Smk,Drk,Educ,HIV,SES
CKD/Alb/low eGFR	Sex,Site,Age,DM,HIV,HTP,Educ,Smk,SES,BMI
CVD	Sex,Site,Age
DM	Sex,Site,Age,SES,Educ,BMI,Drk,HIV
Drk	Sex,Site,Age
Educ	Sex,Site,Age
HDL	Sex,Site,Age,SES,BMI,Smk,HIV,Drk,HTP,Educ,CKD,LDL,DM
HIV	Sex,Site,Age,SES,Educ,Smk,Drk
HTP	Sex,Site,Age,DM,BMI,HIV,SES,Educ,Smk
LDL	Sex,Site,Age,SES,BMI,Smk,HIV,Drk,HTP,Educ,CKD,HDL,DM

SES	Sex,Site,Age,Educ,Drk
Sex	Site,Age
Site	Sex,Age
Smk	Sex,Site,Age,Drk,SES

Defined best set of covariables with DAG

Using the DAG generated for each outcome, we used the algorithm of the 6 step DAG developed by Shrier and Platt[2] to define a minimal set of covariables for one effector and one outcome:

- we defined one effector and outcome, all other variables of the DAG are considered as putative cofactors
- we discarded all putative cofactors that did not validate step 1 of 6-step algorithm DAG: descent of effector in DAG
- for each putative covariable we performed a 6-step DAG
- if no covariable was identified in the 6-step DAG algorithms, we did all combinations of 2 covariables and ran them for each set of algorithms
- if no set of 2 covariables were found in previous step of the 6 steps, we did this for all combinations of 3 covariables, followed by combination of 4, 5, 6 until we obtained one or set of covariables respecting 6-step DAG algorithm
- we computed risk factors (relative risk) of effectors on the outcome using the covariable set defined previously, covariable set used for each couple of outcome/effector is reported in Supplementary Tables 7, 8 and 9.
-

Code source and example found in https://github.com/jeantristanb/dag_6step_epidemiology

[1]Westreich, D. & Greenland, S. The table 2 fallacy: Presenting and interpreting confounder and modifier coefficients. *Am. J. Epidemiol.* 177, 292–298 (2013).

[2]Shrier, I. & Platt, R. W. Reducing bias through directed acyclic graphs. *BMC Med. Res. Methodol.* 8, 70 (2008).

[3]Evans, D., Chaix, B., Lobbedez, T., Verger, C. & Flahault, A. Combining directed acyclic graphs and the change-in-estimate procedure as a novel approach to adjustment-variable selection in epidemiology. *BMC Med. Res. Methodol.* 12, 156 (2012).

[4]Thornley, S., Marshall, R. j, Wells, S. & Rod, J. Using Directed Acyclic Graphs for Investigating Causal Paths for Cardiovascular Disease. *J. Biom. Biostat.* 04, (2013).

[5]Margaritis, D. Learning Bayesian Network Model Structure from Data. (Carnegie-Mellon University, Pittsburgh, PA, 2003).

[6]Scutari, M. Learning Bayesian Networks with the bnlearn R Package. *J. Stat. Software, Artic.* 35, 1–22 (2010).

Supplementary Figure 1

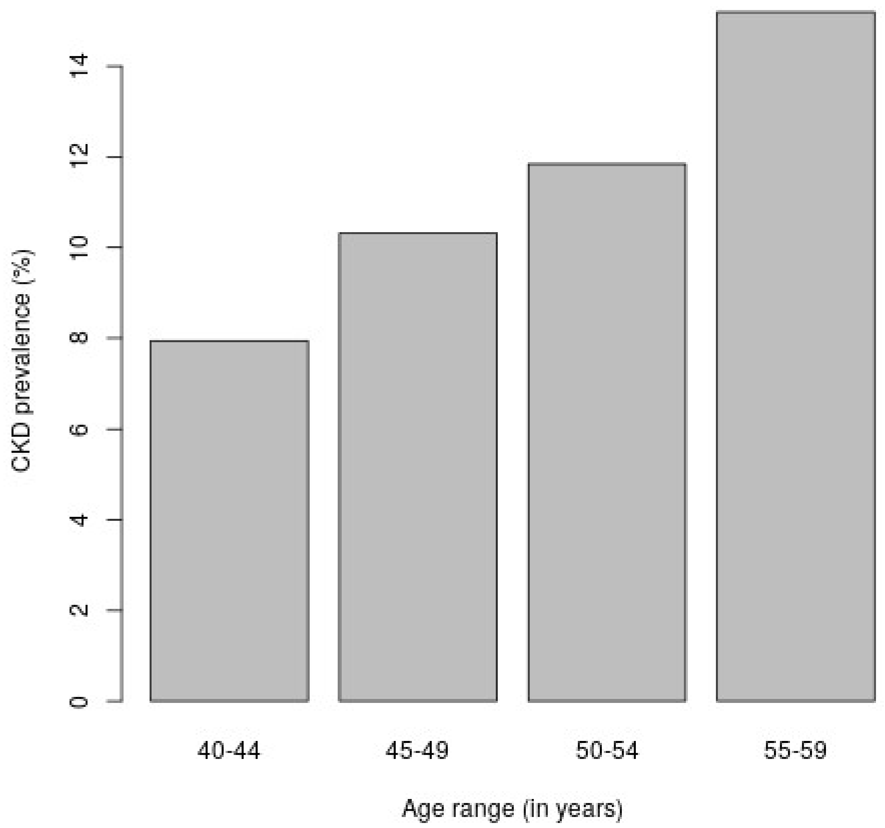

Supplementary Figure 1: Prevalence (%) of CKD in different age groups (Soweto men and women were excluded from this analysis because of incomplete data in the women)

Supplementary Figure 2

No CKD N=6221

CKD N=768

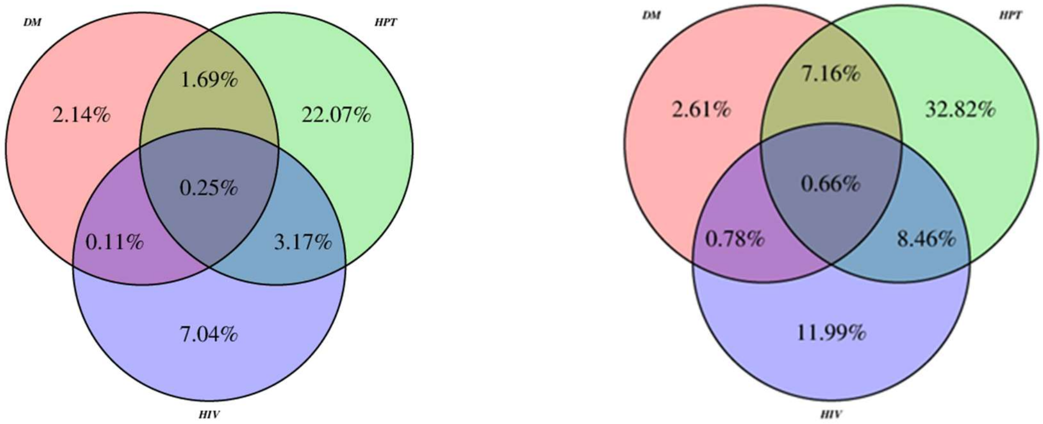

Supplementary Figure 2: Venn diagrams illustrating the associated co-morbidities in the presence chronic kidney disease (CKD) and in its absence (No CKD). Only individuals with complete data were included (all data from Soweto was excluded), explaining the reduced numbers in the dataset. None of the above risk factors but have CKD N= 273, and none of the above risk factors and do not have CKD N=3953

Supplementary Figure 3

A. Low eGFR

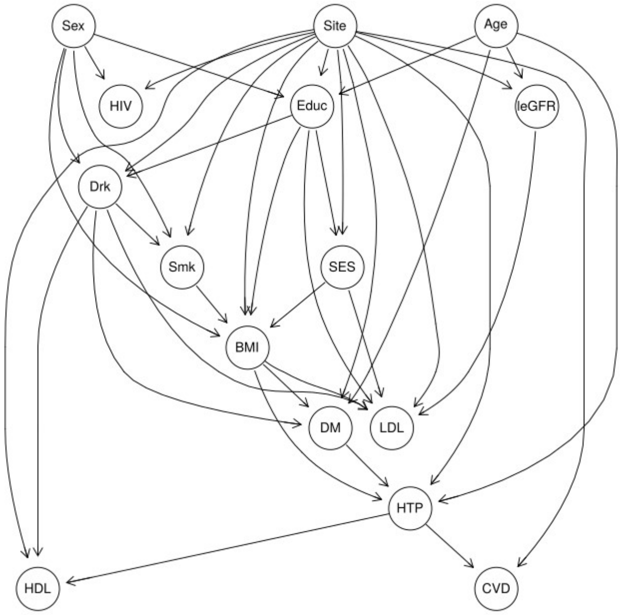

B. Albuminuria

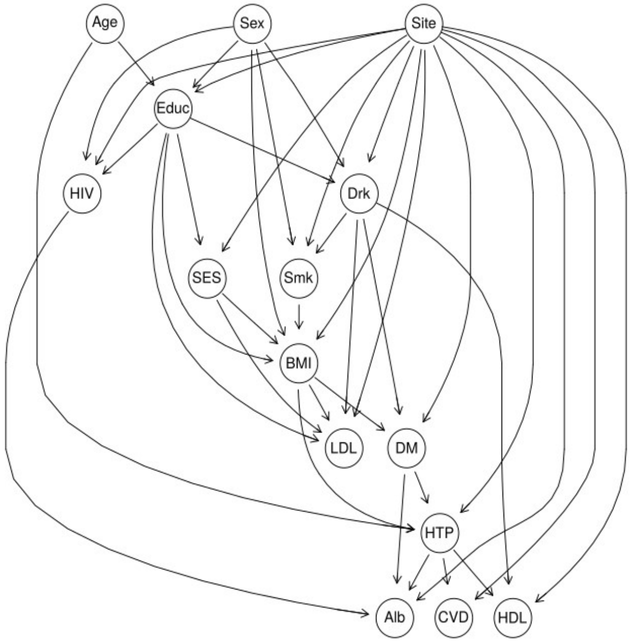

C. CKD

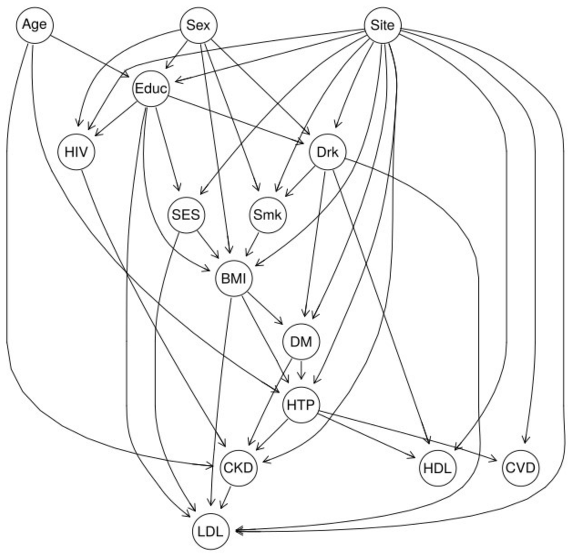

Supplementary Figure 3: Directed Acyclic Graphs (DAG) built with Bayesian network inference using datasets for 3 outcomes: A. Low eGFR B. Albuminuria and C. CKD.

(DM = Diabetes, HTP = hypertension, Drk = Current drinker, Smk = Current Smoker)

Supplementary Tables

Supplementary Table 1: Characteristics of Soweto women with low eGFR and with no indicators of kidney disease

		No indicators of low eGFR	eGFR <60mL/min per 1.73m^2	Totals
**N**		866	40	906
**Age years (sd,N)**		49·04 (5·63,)	50·38 (5·82,)	49·1 (5·64,)
**BMI (kg/m2) (sd,N)**		33·2 (7·26,866)	32·53 (5·54,40)	33·17 (7·19,906)
**Waist circumference cm(sd,N)**		987·64 (143·23,863)	987·85 (135·68,40)	987·65 (142·83,903)
**Education % (n,N)**	**no formal education**	1·1 (2,186)	0 (0,23)	0·3 (2,711)
	**primary**	76·9 (143,186)	91·3 (21,23)	80·5 (572,711)
	**secondary**	22 (41,186)	8·7 (2,23)	19·1 (136,711)
	**tertiary**	0 (0,186)	0 (0,23)	0·1 (1,711)
**SES % (n,N)**	**1**	20 (28,140)	25 (6,24)	23·9 (183,766)
	**2**	46·4 (65,140)	50 (12,24)	46·1 (353,766)
	**3**	21·4 (30,140)	4·2 (1,24)	19·1 (146,766)
	**4**	9·3 (13,140)	20·8 (5,24)	9·8 (75,766)
	**5**	2·9 (4,140)	0 (0,24)	1·2 (9,766)
**HIV % (N)**		21·8 (363)	22·2 (9)	8·9 (372)
**Current smoker % (N)**		5·1 (858)	2·5 (40)	5 (898)
**Hypertension % (N)**		53·9 (866)	70 (40)	54·6 (906)
**Diabetes % (N)**		11·2 (864)	25 (40)	11·8 (904)
**Triglycerides (mol/L)(sd,N)**		0·87 (0·4,866)	1·09 (0·6,40)	0·88 (0·41,906)
**LDL (mmol/l) (sd,N)**		2·97 (0·92,865)	3·33 (0·95,40)	2·98 (0·93,905)
**HDL (mmol/l) (sd,N)**		0·83 (0·48,866)	0·92 (0·54,40)	0·83 (0·49,906)
**Non HDL (sd,N)**		3·36 (0·97,866)	3·82 (0·99,40)	3·38 (0·97,906)
**Cholesterol (mmol/L)(sd,N)**		4·19 (1·09,866)	4·74 (1·07,40)	4·22 (1·09,906)
**Serum Creatinine (µmol/l) (sd,N)**		67·2 (12·71,866)	115·9 (43·84,40)	69·35 (18·37,906)
**CKD-EPI (ml/min1/73m^2) (sd, N)**		91·3 (15·4,866)	50·9 (9·9,40)	89·51 (17·35,906)

Sex	0	0	0	0	0	0	0	0	0	0	0	0	0	0	0

Supplementary Table 2A: Missing data by sex, site and variables in numbers of individuals

Age	All 0	Women 0	Men 0	Agincourt 0	0	Dikgale 0	0	Nairobi 0	0	Nanoro 0	0	Navrongo 0	0	Soweto 0	0
**BMI**	22	15	7	12	6	0	1	0	0	0	1	0	0	1	1
**Waist circumference**	36	22	14	8	6	0	2	3	2	3	1	0	1	2	8
**Sites**	0	0	0	0	0	0	0	0	0	0	0	0	0	0	0
**Education (highest level)**	241	234	7	0	1	0	1	0	0	4	9	5	2	0	219
**SES**	162	160	2	0	0	0	1	0	0	2	5	1	0	0	153
**HIV**	901	698	203	1	1	9	11	82	132	0	0	0	0	61	604
**Current drinker**	2045	1014	1031	0	0	0	3	0	0	4	3	2	2	1025	1006
**Current smoker**	24	16	8	0	2	1	1	0	1	2	4	2	0	2	9
**CVD History**	1016	1013	3	0	0	1	3	0	0	2	4	0	0	0	1006
**Hypertension**	0	0	0	0	0	0	0	0	0	0	0	0	0	0	0
**Diabetes**	194	110	84	27	14	4	17	9	7	12	9	34	30	17	14
**Triglycerides**	230	171	59	49	21	2	9	2	1	16	9	2	3	16	100
**LDL**	334	218	116	57	23	4	17	9	13	20	9	25	18	38	101
**HDL**	230	171	59	49	21	2	9	2	1	16	9	2	3	16	100
**Non HDL**	294	203	91	55	22	2	14	5	5	16	9	20	14	32	100
**Cholesterol**	230	171	59	49	21	2	9	2	1	16	9	2	3	16	100
**Serum Creatinine / eGFR**	230	171	59	49	21	2	9	2	1	16	9	2	3	16	100
**ACR**	2500	1847	653	80	51	74	212	244	165	95	102	203	131	137	1006
**CKD**	2593	1901	692	119	66	76	217	246	166	104	109	204	131	149	1006
**Total (missing and not)**	10703	5895	4808	892	573	356	811	1056	886	1045	1039	1091	923	1025	1006

Table 2B: Comparison of missing data

	Individuals with no missing data	Individuals with missing data*	P value
Current drinker % (N)	42·35 (7221)	40·44 (643)	0·3594#
Diabetes % (N)	5·16 (8039)	5·79 (639)	0·4603#
CVD History % (N)	3·31 (8101)	4·4 (728)	0·1337#
HIV % (N)	12·64 (7885)	14·47 (684)	0·17#
Hypertension % (N)	31·89 (8110)	32·14 (728)	0·9009#
Current smoker % (N)	18·23 (8095)	17·17 (728)	0·514#
Age (years)(SD,N)	49·92 (5·84,8110)	50·33 (5·73,728)	0·0692§
BMI (kg/m^2) (SD,N)	24·16 (6·11,8101)	24·85 (6·26,719)	0·0016§
eGFR ml/min/1.73m^2(SD,N)	98·06 (16·43,8110)	97·9 (14·67,605)	0·0651§
LDL(mmol/L) (SD,N)	2·23 (0·89,8031)	2·3 (0·83,593)	0·0275§
HDL(mmol/L) (SD,N)	1·18 (0·41,8110)	1·18 (0·4,605)	0·8318§
log(ACR) (SD,N)	-1·29 (1·76,8110)	-0·71 (1·93,93)	0·005§
SES (SD,N)	3·29 (1·4,8102)	3·28 (1·4,728)	0·7422§
Waist circumference (cm) (SD,N)	84·09(141·54,8095)	84·85 (143·29,718)	0·1388§

* Individuals with missing data were those that were missing across the study as a whole (but excluding the Soweto women who were not included in the prevalence calculations and individuals for whom serum and urine creatinine measurements were out of range)
# for comparison of % prevalence, p values were calculated using the Fisher exact test
§ for comparison of the mean, p values were calculated using the Wilcoxon test

Supplement Table 3: Mean eGFRs for men and women using the MDRD-4 and the CKD-EPI equations across study sites

	MDRD-4 (Eth)					MDRD-4 (no Eth)				
	**Mean (sd)**		**p.value sex**	**Sex*Site models interaction**		**Mean (sd)**		**p.value sex**	**Sex*Site models interaction**	
**Site**	Men	Women		B Site (pvalue)	B sex:site (Pvalue)	Men	Women		B Site (pvalue)	B sex:site (P value)
**Agincourt**	123·9 (35·98)	103·22 (25·2)	<0·0001	-1·6e+01 (<0·0001)	11·63 (<0·0001)	102·23 (29·69)	85·16 (20·79)	<0·0001	-1·3e+01 (<0·0001)	9·60 (<0·0001)
**Dikgale**	123·67 (41·17)	106·52 (38·35)	<0·0001	-1·3e+01 (<0·0001)	8·10 (0·005)	102·04 (33·97)	87·89 (31·65)	<0·0001	-1·1e+01 (<0·0001)	6·68 (0·005)
**Soweto**	127·2 (31·84)	101·39 (31·31)	<0·0001	-1·8e+01 (<0·0001)	16·75 (<0·0001)	104·95 (26·27)	83·66 (25·83)	<0·0001	-1·5e+01 (<0·0001)	13·82 (<0·0001)
**Nairobi**	127·11 (41·95)	111 (34·64)	<0·0001	-8·4e+00 (<0·0001)	7·06 (0·0025)	104·87 (34·61)	91·58 (28·58)	<0·0001	-6·9e+00 (<0·0001)	5·82 (0·0025)
**Navrongo**	123·75 (37·44)	108·91 (27·23)	<0·0001	-1·1e+01 (<0·0001)	5·78 (0·011)	102·1 (30·89)	89·86 (22·46)	<0·0001	-8·7e+00 (<0·0001)	4·77 (0·0111)
**Nanoro**	128·47 (32·62)	119·42 (33·73)	<0·0001			106 (26·91)	98·53 (27·83)	<0·0001		
			Sex	9·05 (<0·0001)				Sex	7·47 (<0·0001)	

	CKD-EPI (Eth)					CKD-EPI (No Eth)				
**Site**	**Mean (sd)**		**p.value sex**	**Sex*Site models interaction**		**Mean (sd)**		**p.value sex**	**Sex*Site models interaction**	
**Agincourt**	Men	Women		B Site (pvalue)	B sex:site (P value)	Men	Women		B Site (pvalue)	B sex:site (P value)
**Dikgale**	115·44 (19·21)	105·51 (18·72)	<0·0001	-1·0e+01 (<0·0001)	6·75 (<0·0001)	99·6 (16·57)	91·04 (16·15)	<0·0001	-8·9e+00 (<0·0001)	5·83 (<0·0001)
**Soweto**	115·49 (18·71)	105·58 (20·96)	<0·0001	-1·0e+01 (<0·0001)	6·74 (<0·0001)	99·64 (16·14)	91·1 (18·09)	<0·0001	-8·9e+00 (<0·0001)	5·81 (<0·0001)
**Nairobi**	118·23 (17·73)	103·74 (20·11)	<0·0001	-1·2e+01 (<0·0001)	11·32 (<0·0001)	102·01 (15·3)	89·51 (17·35)	<0·0001	-1·0e+01 (<0·0001)	9·77 (<0·0001)
**Navrongo**	118·34 (18·24)	110·7 (20·74)	<0·0001	-5·2e+00 (<0·0001)	4·47 (0·0005)	102·11 (15·73)	95·52 (17·89)	<0·0001	-4·5e+00 (<0·0001)	3·86 (0·0005)
**Nanoro**	115·38 (19·23)	109·35 (17·26)	<0·0001	-6·5e+00 (<0·0001)	2·87 (0·0222)	99·55 (16·59)	94·35 (14·89)	<0·0001	-5·6e+00 (<0·0001)	2·47 (0·0222)
**Site**	119·03 (17·17)	115·86 (16·29)	<0·0001			102·7 (14·81)	99·97 (14·05)	<0·0001		
			Sex	3·17 (0·00023)				Sex	2·74 (0·00023)	

Mean (sd) (mls/min/1.73m^2) for each site, and sex (M=men, W=women)

"p.value sex" is the result (p value) of statistical comparison between men and women (t test)

"**Sex*Site models interaction**" is result of generalized linear models for sex, site interaction with Nanoro as reference

"B site" is values of B (p value) for eGFR values site increase under Nanoro

"B sex: site" is B and p value of sex at each site, Sex is the general term (B,(p value)) for sex

Supplementary Table 4: Low eGFR: % (CI 95%) for MDRD-4 and CKD-EPI equations by site, sex and All

		MDRD-4		CKD-EPI	
**Site**	**Sex**	**Ethnicity Factors**	**No Ethnicity Factors**	**Ethnicity Factors**	**No Ethnicity Factors**
**All**	**W**	1·84 (1·49-2·26)	7·39 (6·68-8·16)	1·35 (1·05-1·72)	3·43 (2·94-3·99)
	**M**	1·36 (1·04-1·78)	3·3 (2·79-3·91)	1·29 (0·98-1·69)	2·19 (1·77-2·69)
	**All**	1·62 (1·37-1·91)	5·52 (5·07-6·02)	1·32 (1·1-1·58)	2·86 (2·53-3·23)
**Agincourt**	**W**	2·07 (1·23-3·41)	6·99 (5·34-9·08)	1·68 (0·94-2·94)	4·14 (2·89-5·86)
	**M**	1·38 (0·61-2·95)	3·35 (2·03-5·42)	1·38 (0·61-2·95)	2·37 (1·29-4·21)
	**All**	1·8 (1·17-2·73)	5·55 (4·39-6·98)	1·56 (0·98-2·45)	3·44 (2·54-4·63)
**Dikgale**	**W**	2·19 (1·22-3·81)	10·77 (8·45-13·62)	1·85 (0·98-3·39)	4·04 (2·66-6·04)
	**M**	1·07 (0·28-3·36)	2·5 (1·1-5·3)	1·07 (0·28-3·36)	1·79 (0·66-4·35)
	**All**	1·83 (1·09-3·02)	8·12 (6·44-10·19)	1·6 (0·91-2·74)	3·32 (2·27-4·79)
**Soweto**	**W**	2·43 (1·56-3·71)	11·26 (9·31-13·54)	1·43 (0·8-2·51)	4·42 (3·21-6·02)
	**M**	1·26 (0·66-2·31)	2·85 (1·89-4·25)	1·03 (0·5-2·01)	1·83 (1·08-3·02)
	**All**	1·85 (1·3-2·62)	7·13 (6-8·45)	1·23 (0·79-1·9)	3·14 (2·4-4·09)
**Nairobi**	**W**	2·59 (1·65-4)	8·64 (6·84-10·85)	1·85 (1·08-3·11)	4·32 (3·07-6·02)
	**M**	1·11 (0·52-2·27)	3·33 (2·19-4·99)	1·11 (0·52-2·27)	1·53 (0·81-2·8)
	**All**	1·9 (1·3-2·75)	6·14 (5·02-7·5)	1·5 (0·98-2·28)	3·01 (2·23-4·02)
**Navrongo**	**W**	1·47 (0·82-2·56)	4·62 (3·38-6·27)	1·13 (0·57-2·14)	2·37 (1·51-3·66)
	**M**	2·02 (1·2-3·33)	4·29 (3·04-6·01)	2·02 (1·2-3·33)	3·16 (2·1-4·69)
	**All**	1·73 (1·18-2·5)	4·47 (3·55-5·6)	1·55 (1·03-2·29)	2·74 (2·03-3·67)
**Nanoro**	**W**	0·54 (0·2-1·33)	3·33 (2·31-4·75)	0·43 (0·14-1·18)	1·72 (1·02-2·84)
	**M**	1·17 (0·62-2·15)	3·08 (2·11-4·45)	1·06 (0·54-2·01)	2·23 (1·42-3·45)
	**All**	0·86 (0·51-1·42)	3·21 (2·48-4·14)	0·75 (0·43-1·29)	1·98 (1·42-2·74)

Supplement Table 5: Percentage distribution of socio-economic-status (SES) and education by sex and sites

		Agincourt		Digkale		Nairobi		Nanoro		Navrongo		Soweto	
		Men	Women	Men	Women	Men	Women	Men	Women	Men	Women	Men	Women
**SES**	1	20·3	11·8	20·0	10·3	11·0	12·3	13·6	19·7	15·7	19·4	2·7	23·9
	2	26·2	22·0	24·6	20·4	19·2	24·0	20·2	18·2	16·2	19·5	9·5	46·1
	3	12·0	12·7	11·4	14·3	22·8	22·8	18·6	19·7	17·6	19·7	14·8	19·1
	4	23·7	23·5	18·2	22·9	18·5	23·0	17·1	20·1	24·4	23·6	25·8	9·8
	5	17·8	30·0	25·7	32·0	28·6	17·9	30·4	22·4	26·3	17·8	47·1	1·2
**Education (highest level)**	no formal education	21·7	30·4	5·4	9·4	4·0	10·4	73·7	93·8	61·3	78·5	0·8	0·3
	primary	40·8	38·2	33·9	33·8	49·9	62·2	16·6	5·1	23·2	15·3	10·7	80·5
	secondary	30·8	26·1	56·1	54·0	43·6	26·9	8·0	0·9	13·3	5·4	73·3	19·1
	tertiary	6·7	5·3	4·6	2·7	2·5	0·5	1·7	0·2	2·3	0·8	15·2	0·1

Supplementary Table 6: Risk factors computed with relative risk and co variables chosen with 6-step DAG algorithm for albuminuria and for low eGFR

Alb	Effectors	Agincourt	Dikgale	Nairobi	Nanoro	Navrongo	Soweto (Men)
	**Male sex**	0·86 (0·63-1·17)	0·73 (0·45-1·13)	0·85 (0·61-1·18)	1·02 (0·69-1·50)	1·01 (0·69-1·48)	-
	**Age**	1·03 (1·00-1·05)	1·04* (1·00-1·07)	1·03 (1·00-1·06)	1·03* (1·00-1·07)	1·01 (0·98-1·05)	1·04* (1·00-1·07)
	**BMI**	1·00 (0·98-1·03)	1·00 (0·97-1·02)	1·01 (0·98-1·04)	0·97 (0·91-1·03)	0·99 (0·94-1·04)	1·03 (0·99-1·06)
	**Diabetes**	2·11** (1·30-3·27)	2·40*** (1·41-3·91)	2·29*** (1·39-3·57)	3·08*** (1·49-5·71)	2·59 (0·79-6·26)	2·85*** (1·61-4·78)
	**Highest level of education**	1·02 (0·85-1·22)	0·99 (0·74-1·33)	0·98 (0·74-1·29)	0·91 (0·59-1·31)	1·04 (0·79-1·33)	0·66* (0·47-0·95)
	**HIV**	1·63** (1·20-2·20)	1·50 (0·93-2·34)	3·05# (2·08-4·40)	-	-	1·38 (0·86-2·13)
	**Hypertension**	1·39* (1·02-1·91)	2·02*** (1·34-3·09)	2·44# (1·75-3·40)	2·03** (1·30-3·10)	3·04# (2·09-4·42)	3·76# (2·30-6·47)
	**SES**	0·99 (0·89-1·10)	1·03 (0·89-1·19)	0·85** (0·75-0·96)	1·18* (1·02-1·36)	1·05 (0·92-1·21)	0·97 (0·81-1·18)
	**Current smoker**	0·86 (0·47-1·52)	0·32** (0·14-0·71)	1·57 (0·94-2·55)	2·15* (1·12-3·88)	1·05 (0·61-1·78)	0·95 (0·63-1·43)
	**Current drinker**	1·53* (1·01-2·27)	0·93 (0·53-1·57)	1·69* (1·11-2·52)	1·23 (0·82-1·90)	0·92 (0·62-1·38)	-
	**CVD History**	0·62 (0·22-1·36)	1·24 (0·55-2·40)	1·54 (0·73-2·85)	1·75 (0·43-4·68)	1·04 (0·26-2·77)	-
	**N**	1261	844	1356	1850	1630	825
**Low eGFR**	**Effectors**	**Agincourt**	**Dikgale**	**Nairobi**	**Nanoro**	**Navrongo**	**Soweto (All)**
	**Male sex**	0·58 (0·28-1·09)	0·48 (0·16-1·16)	0·31** (0·13-0·64)	1·27 (0·67-2·48)	1·55 (0·85-2·87)	0·76 (0·34-1·79)
	**Age**	1·14# (1·07-1·22)	1·10** (1·03-1·18)	1·10** (1·04-1·16)	1·08* (1·02-1·14)	1·11*** (1·05-1·18)	1·04 (0·97-1·11)
	**BMI**	1·05* (1·00-1·08)	1·02 (0·97-1·06)	0·98 (0·92-1·03)	1·02 (0·92-1·11)	0·98 (0·89-1·06)	1·02 (0·96-1·07)
	**Diabetes**	1·15 (0·34-2·87)	1·80 (0·60-4·43)	2·55* (1·03-5·49)	2·10 (0·50-5·85)	0	1·89 (0·55-5·03)
	**Highest level of education**	1·08 (0·75-1·53)	1·31 (0·76-2·32)	0·67 (0·40-1·12)	1·21 (0·63-1·99)	1·41 (0·96-1·98)	1·35 (0·72-2·53)
	**HIV**	1·58 (0·86-2·86)	2·84** (1·28-6·07)	1·60 (0·68-3·35)	-	-	0·80 (0·23-2·16)
	**Hypertension**	2·74** (1·36-6·11)	1·10 (0·51-2·36)	1·63 (0·83-3·13)	2·85** (1·42-5·51)	1·31 (0·67-2·45)	0·83 (0·37-1·87)
	**SES**	1·08 (0·88-1·35)	1·09 (0·83-1·45)	0·85 (0·67-1·09)	0·96 (0·75-1·21)	1·01 (0·81-1·27)	1·05 (0·73-1·55)
	**Current smoker**	0·39 (0·06-1·27)	0·44 (0·11-1·26)	0·39 (0·06-1·27)	2·28 (0·78-5·37)	2·63** (1·40-4·82)	0·89 (0·33-2·34)
	**Current drinker**	0·52 (0·18-1·20)	0·90 (0·35-2·03)	0·54 (0·16-1·35)	0·90 (0·47-1·79)	1·41 (0·74-2·85)	-
	**CVD History**	1·82 (0·55-4·53)	0	0·60 (0·03-2·77)	0	3·10 (0·75-8·55)	-
	**N**	1261	844	1356	1850	1630	1178

Legend supplementary table 6: Excluded: HIV in Nanoro and Navrongo due to low prevalence; CVD History and Current Drinker for Soweto

For albuminuria risk factors computed only for men for Soweto. N is sample number used to compute risk factors.

“0” values observed for low eGFR : when too few individuals have low eGFR and effectors/risk factors

- *P ≤ 0.05
- ** P≤0.01
- ***p≤0.001
- #P≤0.0001

Supplementary Table 7: Covariables defined with "6-step DAG" used to compute relative risk for each outcome and each effectors

Effectors	CKD	Albuminuria	Low eGFR
**Age**	Site,Sex	Site,Sex	Site,HIV,Sex
**Site**	Age,Sex	Age,Sex	Age,Sex
**BMI**	HIV,Age,Site,Sex,Drk,Smk,Educ	HIV,Age,Site,Sex,Drk,Smk,Educ	Age,Site
**Current drinker**	Site,Sex,Educ	Site,Sex,Educ	Age,Site
**Current smoker**	Site,Sex,Drk	Site,Sex,Drk	Age,Site
**CVD History**	Site,HTP	Site,HTP	Age,Site,HTP
**Diabetes**	BMI,Site,Drk	BMI,Site,Drk	Age,Site
**Highest level of education**	Age,Site,Sex	Age,Site,Sex	Age,Site
**HIV**	Site,Sex,Educ	Sex,Educ,DM,Site,HTP	Site
**Hypertension**	BMI,Age,DM,Site,HIV	DM,HIV,Site	Age,Site
**SES**	Site,Educ	Site,Educ	Age,Educ,Site
**Sex (Men)**	Age,Site	Age,Site	Age,Site
**Site**	Age,Sex	Age,Sex	Age,Sex

(DM = Diabetics, Smk = Current smoker, HTP = Hypertension, Drk = Current Drinker)

Supplementary Table 8: Covariables defined with "6-step DAG" algorithm to compute relative risk for each outcome, sites and each effectors. Covariable lists have been grouped by site and outcome.

Effectors	Sites list	Covariable used for each Outcome		
		CKD	Alb	Low eGFR
**Age**	Agincourt;Dikgale;Nairobi			HIV,Sex
**Age**	Agincourt;Dikgale;Nairobi;Nanoro;Navrongo	Sex	Sex	
**Age**	Nanoro; Navrongo			Sex
**Age**	Soweto	Smk	Smk	Smk,HIV,Sex
**BMI**	Agincourt;Dikgale;Nairobi	HIV,Age,Sex,Drk,Smk,Educ	HIV,Age,Sex,Drk,Smk,Educ	
**BMI**	Agincourt;Dikgale;Nairobi;Nanoro;Navrongo; Soweto			Age
**BMI**	Nanoro; Navrongo	Age,Drk	Age,Drk	
**BMI**	Soweto	Educ	Educ	
**Current drinker**	Agincourt;Dikgale;Nairobi;Nanoro;Navrongo	Sex,Educ	Sex,Educ	Age
**Current smoker**	Agincourt;Dikgale;Nairobi;Nanoro;Navrongo	Sex,Drk	Sex,Drk	Age
**Current smoker**	Soweto	Age,Educ,SES,HIV	Age,Educ,SES,HIV	Age,HIV,Sex
**CVD History**	Agincourt;Dikgale;Nairobi;Nanoro;Navrongo	HTP	HTP	Age,HTP
**Diabetes**	Agincourt;Dikgale;Nairobi	BMI,Drk	BMI,Drk	
**Diabetes**	Agincourt;Dikgale;Nairobi;Nanoro;Navrongo; Soweto			Age
**Diabetes**	Nanoro; Navrongo	Age,BMI,Drk	Age,BMI,Drk	
**Diabetes**	Soweto	BMI	BMI	
**Highest level of education**	Agincourt;Dikgale;Nairobi;Nanoro;Navrongo	Age,Sex	Age,Sex	
**Highest level of education**	Agincourt;Dikgale;Nairobi;Nanoro;Navrongo; Soweto			Age
**Highest level of education**	Soweto	Age	Age	
**HIV**	Agincourt;Dikgale;Nairobi	Sex,Educ	Sex,DM,Educ,HTP	Age,Sex
**HIV**	Soweto	Educ	Educ	Age,Smk,Sex
**Hypertension**	Agincourt;Dikgale;Nairobi	Age,,DM,,BMI,,HIV	DM,HIV	
**Hypertension**	Agincourt;Dikgale;Nairobi;Nanoro;Navrongo; Soweto			Age
**Hypertension**	Nanoro; Navrongo	DM,Age	DM	
**Hypertension**	Soweto	Age,DM,Educ,BMI,HIV	DM,Educ,HIV	
**SES**	Agincourt;Dikgale;Nairobi;Nanoro;Navrongo; Soweto	Educ	Educ	Age, Educ
**Sex (Men)**	Agincourt;Dikgale;Nairobi;Nanoro;Navrongo	Age	Age	
**Sex (Men)**	Agincourt;Dikgale;Nairobi;Nanoro;Navrongo; Soweto			Age

(DM = Diabetics, Smk = Current smoker, HTP = Hypertension, Drk = Current Drinker, Educ=education)
